# Subjective sensory sensitivity and its relationship with anxiety in people with probable migraine

**DOI:** 10.1111/head.14219

**Published:** 2021-10-20

**Authors:** Alice Price, Petroc Sumner, Georgina Powell

**Affiliations:** ^1^ School of Psychology Cardiff University Cardiff UK; ^2^ Cardiff University Brain Research Imaging Centre (CUBRIC), School of Psychology Cardiff University Cardiff UK

**Keywords:** anxiety, migraine, sensory processing, sensory sensitivity, sensory threshold

## Abstract

**Objective:**

To better characterize differences in interictal sensory experience in adults with migraine and more comprehensively describe the relevance of anxiety to these experiences.

**Background:**

Evidence suggests that sensitivity to sensory input may not be limited to migraine attacks but continues between them. However, there is a need to better understand whether this is the case across senses, and to clearly distinguish sensory experience from measured sensory threshold, which are not straightforwardly related. Previous literature also indicates a co‐occurrence between sensory sensitivity, migraine, and anxiety, but this relationship remains to be fully elucidated.

**Methods:**

The present cross‐sectional study used online questionnaires to investigate how self‐reported sensory experiences relate to migraine in a large community sample including 117 individuals with probable migraine and 827 without. Mediation analyses were also used to determine whether any relationship between migraine and sensory sensitivity was mediated by anxiety.

**Results:**

Significant increases in subjective reports of sensory sensitivity (*d* = 0.80) and sensory avoidance (*d* = 0.71) were found in participants with migraine. Anxiety symptoms partially mediated the relationship between subjective sensory sensitivity and migraine. Finally, visual, movement, and auditory subscales were found to provide unique explanatory variance in analyses predicting the incidence of migraine (area under the curve = 0.73, 0.69, 0.62 respectively).

**Conclusion:**

Subjective sensory sensitivities are present between attacks and across senses in individuals with migraine. Anxiety symptoms are relevant to this relationship; however, sensory sensitivities appear to exist independent of this affective influence. The implications of interictal sensitivities for the daily lives of those with migraine should, therefore, be considered in clinical management wherever appropriate.

**Abbreviations**
AASP: Adolescent/Adult Sensory Profile
HADS: Hospital Anxiety and Depression Scale
MSQ: Migraine Screening Questionnaire

## BACKGROUND

Migraine attacks are typically characterized by enduring headache, nausea and sensitivity to light, sound, and odors.^1^ Sensory stimuli can also trigger or worsen attacks,^2^, ^3^ and sensory disturbances (most often visual) commonly occur in those who experience migraine with aura.^4^

In this paper, we investigate whether differences in sensory experience exist in people with migraine *between* attacks (also known as interictal differences). It is important to distinguish two meanings of “sensitivity”: heightened sensory experience or measured thresholds for detecting or discriminating sensory stimuli. These two meanings are not straightforwardly related.

There is some evidence that between attacks, people with migraine show a different threshold sensitivity to stimuli compared with people without migraine. The evidence is mixed; for example, both higher and lower sensory thresholds have been reported across modalities.^5^ However, threshold performance does not predict the strength or quality of self‐reported sensory experience,^6^, ^7^, ^8^ highlighting a need to clearly distinguish between these two concepts. Threshold measurements are thought to tap the basic capabilities of early processing, whereas the subjective experience involves extensive activation and feedback well beyond the primary sensory cortices.^6^ Given that the subjective sensory experience is associated with reduced well‐being^7^, ^8^ and anxiety,^9^ understanding whether sensory experiences differ in migraine remains important whether or not threshold differences are confirmed.

Evidence from children and adolescents with migraine has shown increases in self‐ or parent‐reported sensory behaviors, which may indicate heightened sensitivity to sensory information (known as sensory hypersensitivity^10^, ^11^). In children, this reported hypersensitivity was associated with reductions in quality of life.

Furthermore, work investigating the presence of psychotic symptoms and hallucinations in migraine has also found evidence of hypersensitivity.^12^ Although the self‐report measure used in this study was not a direct measure of sensory sensitivity, people with migraine more frequently endorsed items relating to a heightened experience of sensory stimuli when compared with people without migraine.

Finally, in an investigation of the relationship between subjective sensory sensitivity and attention in migraine, Leveque et al.^13^ recently found that adults with migraine self‐reported increased sensitivity to light, sounds, and odors between attacks when compared with people without migraine. Sensory sensitivities were correlated with self‐reported attentional difficulties, but not migraine disability.

Taken together, this literature is consistent with the idea that migraine is associated with interictal differences in subjective sensory experience. However, an additional consideration in investigating sensory experience in migraine is the experience of anxiety. Anxiety is found to commonly co‐occur with migraine at both trait and clinical levels,^14^, ^15^, ^16^, ^17^ and the two conditions might share genetic predispositions,^18^ neurotransmitter systems,^19^ and psychological influences (e.g., interoceptive conditioning^20^). Anxiety is also associated with sensory hypersensitivity in people without migraine.^9^ It is therefore possible that if differences in sensory experience exist in migraine, they could be driven (at least in part) by heightened levels of anxiety.

It is worth noting that Leveque et al.^13^ did not find that anxiety explained differences in sensory processing that they observed. In fact, they did not find that anxiety and sensory sensitivities were correlated at all in their sample. Therefore, the triadic relationship that may exist between migraine, sensory experience, and anxiety has not been well characterized in the literature so far and could be addressed by using more formal mediation analyses with a larger pool of participants. Given differences in anxiety and subjective sensory sensitivity could have relevance for the day‐to‐day experiences of individuals with migraine, these effects are worth being fully explored.

In the present study, our aim was therefore to better characterize differences in interictal sensory experience in migraine. We did this by (1) using an established questionnaire in sensory processing that is underpinned by theory and spans the range of sensory modalities, (2) describing the relationship with anxiety more comprehensively using a formal mediation model, (3) exploring the individual and unique contribution of different sensory modalities, and (4) using a large community sample of 117 individuals with migraine and 827 comparison participants without migraine.

The sensory experience questionnaire we used was the Adolescent/Adult Sensory Profile (AASP^21^). The AASP was the most appropriate for our study; unlike other similar measures, it is designed for general population use and provides a measure of subjective sensory experience across six sensory modalities (taste/smell, visual, auditory, tactile, movement, and activity). We were interested in two subscales of the AASP that indicate subjective sensory hypersensitivity: sensory sensitivity and sensory avoidance. Despite its common use in sensory processing literature, the AASP is yet to be used in adult migraine populations, the absence of which has been noted.^22^ To explore the relationship between sensory experience, migraine, and anxiety, we also collected data on anxiety, using the Hospital Anxiety and Depression Scale (HADS).^23^

We hypothesized that migraine would be associated with increased subjective sensory sensitivity and avoidance across all modalities, and this relationship would be mediated by symptoms of anxiety. We speculated that vision might be the dominant sense driving these relationships, because it is commonly associated with migraine aura and triggers.^2^, ^4^

## METHOD

### Participants

Participants were recruited from the community via two methods. The first involved emailing participants from a community health list with an advertisement to participate in a survey. The advert described the broad interest in dizziness (the findings of which relate to another study^24^), sensory sensitivity, and migraine held by the researchers, while emphasizing the desire for a range of participants regardless of experience with topics of interest, excluding only those under 18. Approximately 2500 responses were received (of 18,683 email addresses used); 465 participants had missing data for the AASP, while 1379 had missing anxiety data. Analyses therefore only included those with complete data for all measures of interest (*n* = 818). Participants were aged between 19 and 86 (mean = 57.0, SD = 13.8) and 604 (74%) were female. Median‐reported education attainment was 3, where 0 = no education, 1 = General Certificate of Secondary Education/O Level, 2 = A‐level/Business and Technology Education Council, 3 = Undergraduate, and 4 = Postgraduate.

The second recruitment method used the website Prolific Academic, on which the public can participate in surveys and receive compensation. Participants were compensated £5 for the survey. Of 214 responses received, 14 had missing AASP data, while 74 had missing anxiety data. A total of 126 participants returned valid and complete responses for each measure and were therefore included in analyses. Participants were aged between 18 and 54 (mean = 26.8, SD = 6.8), and 35 (28%) were female. Median educational attainment was 3.

The final combined sample therefore consisted of 944 participants aged between 18 and 86 (mean = 53.0, SD = 16.6), 639 of which were female (68%). Cardiff University's School of Psychology ethics committee provided approval for all procedures. Participants read a consent form online, before providing electronic informed consent via an on‐screen tick box.

This is an a priori secondary analysis of collected data, which was primarily analyzed to answer questions concerning visually induced dizziness.^24^, ^25^ The sample size was based on available data; no statistical power calculation was conducted prior. All data reported in this article will be made available following acceptance.

### Measures

All questionnaires were delivered online via Qualtrics. Demographic information and details of currently diagnosed vestibular disorder were collected.

#### Migraine Screening Questionnaire^26^

The Migraine Screening Questionnaire (MS‐Q) includes five items, which ask individuals about migraine episodes experienced in their lifetime, each with a yes/no response. Participants reporting four or more “yes” responses were categorized as having probable migraine. Example items include “Do you usually suffer from nausea when you have a headache?” and “Does light or noise bother you when you have a headache?” The MS‐Q shows adequate validity and reliability (Cronbach's *α* = 0.82).^26^

#### Adolescent/Adult Sensory Profile^27^

The AASP is a 60‐item self‐report measure of sensory function as it relates to Dunn's model.^28^ Of four possible subscales, we were only interested in the sensory sensitivity and sensory avoidance subscales. Both subscales are argued to indicate subjective sensory sensitivity but refer to different behavioral reactions to sensory input. Although the sensory sensitivity subscale represents a dislike for sensory stimuli and distractibility in its presence (e.g., “I’m uncomfortable wearing certain fabrics”), sensory avoidance indicates behaviors which limit exposure to stimuli and restrict unpredictability (e.g., “I avoid or wear gloves during activities that will make my hands messy”). Higher subscale scores indicate greater levels of the corresponding sensory behavior. The AASP quadrants have been found to have moderate to good internal consistency (Cronbach's *α* between 0.66 and 0.81) and construct validity.^21^, ^27^

Subscales remained separate for initial analyses; however, for ease of interpretation and due to their high collinearity (*r* = 0.78), sensory sensitivity and sensory avoidance subscales were combined into a single variable (referred to as “subjective sensory sensitivity”) for mediation analysis.

Items of the AASP assess sensory processing across six modalities: taste/smell, visual, auditory, tactile, movement, and activity. As in previous work,^29^, ^30^ modality‐specific subscales were also calculated to explore the relative influence of sensory sensitivities in each domain on migraine. This involved summing items from both sensory avoidance and sensory sensitivity subscales according to their associated modality.

#### Hospital Anxiety and Depression Scale^23^

The HADS is a 14‐item measure assessing the symptoms of depression and anxiety. Individuals are asked to indicate the frequency with which they experience each item, on a four‐point scale (e.g., where 0 = Not at all, 1 = Occasionally, 2 = A lot of the time, 3 = Most of the time). Given the overlapping literature between migraine, sensory processing, and anxiety, we focused our analysis on the seven‐item anxiety subscale (e.g., “Worrying thoughts go through my mind”).

### Statistical analyses

Descriptive statistics were calculated, including frequencies and means for all measures of interest. Relevant parametric assumptions were confirmed using visualizations, kurtosis and skewness values, and Levene's test for homogeneity of variances.

Two‐tailed, between‐subjects, independent *t*‐tests were conducted to determine whether participants with and without migraine significantly differed in their sensory sensitivity, sensory avoidance, and HADS‐A scores, before these variables were entered into mediation analyses. As described, sensory sensitivity and sensory avoidance subscales were then combined into a single variable (“subjective sensory sensitivity”) for mediation analysis.

Mediation analysis is a statistical approach, which seeks to clarify whether the effect of an independent variable on a dependent variable occurs via a third, mediating variable. Mediation can either be complete or partial. Complete mediation would suggest that the independent variable (in this case, subjective sensory sensitivity) has no direct effect on the dependent variable (migraine), and the entire effect occurs indirectly via the mediating variable (anxiety). Partial mediation instead indicates both a direct effect (e.g., of subjective sensory sensitivity on migraine) and an indirect effect (e.g., of subjective sensory sensitivity on anxiety, which in turn influences migraine). In this analysis, we will determine to what extent anxiety symptoms mediate the relationship between subjective sensory sensitivity and migraine, controlling for age and gender. Mediation analyses were conducted using model four of the PROCESS macro^31^ in SPSS 25.0,^32^ using bootstrapping with 5000 samples and 95% confidence intervals. Indirect effects were deemed to be significant if corresponding confidence intervals do not contain zero.^33^

Importantly, mediation analysis does not in itself imply causal relationships unless an experimental design which manipulates variables is used. This study was instead cross‐sectional, and relationships are therefore correlational. Mediation analyses using each subscale separately also found an identical pattern of results (available in the Supporting Information).

Subsequent exploratory analyses used between‐subjects *t*‐tests to ascertain whether those with migraine significantly differed in their scores on the six modality subscales derived from the AASP. Bivariate Pearson correlations were also calculated to determine the degree of collinearity between the subscales. The predictive ability of each modality upon migraine was determined individually using logistic regression, before all six were entered into a multiple logistic regression model to establish their unique contributions. Anxiety was also included to control for its influence. Relevant assumptions of logistic regression were assessed, and the Hosmer and Lemeshow goodness‐of‐fit test was calculated.

Finally, following interpretation of our initial mediation model, a post hoc mediation analysis was conducted to determine whether depression symptoms, also measured by the HADS, mediated the relationship between subjective sensory sensitivity and migraine. Details of this analysis, which found no mediating effect of depression symptoms, are available in the Supporting Information.

Significance levels were specified as *p* < 0.05 for all analyses.

## RESULTS

### Did people with probable migraine report higher sensitivity, avoidance, and anxiety?

**FIGURE 1:**
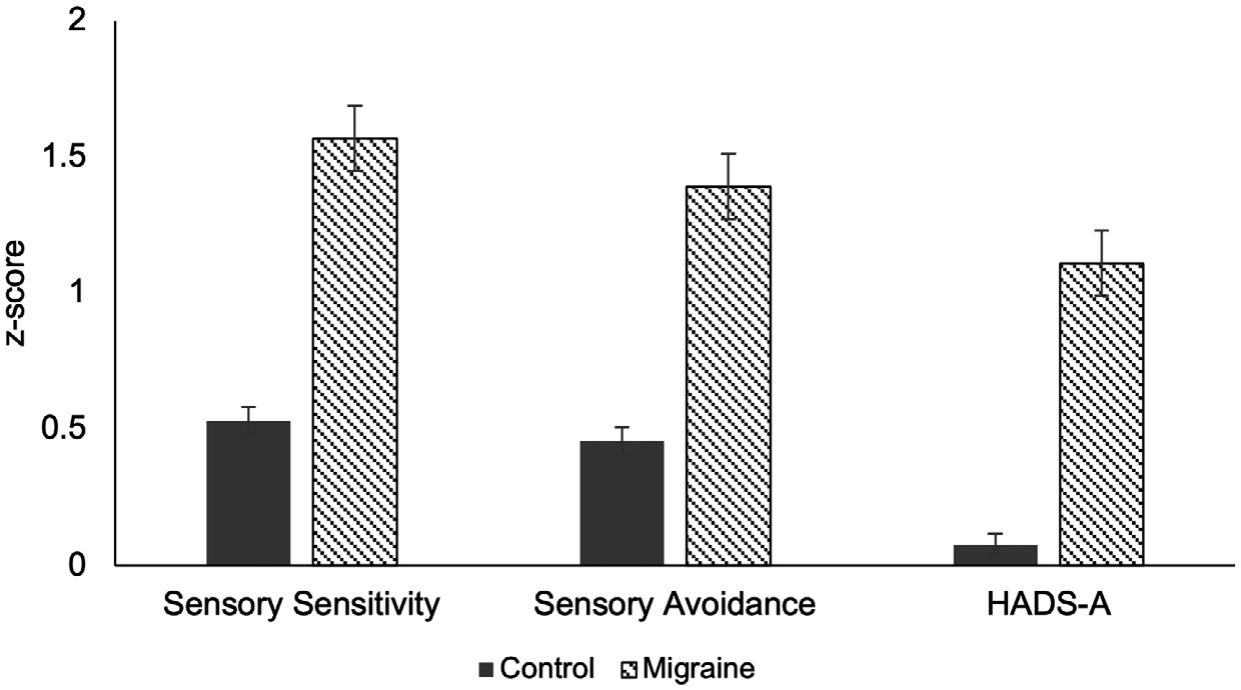
Mean *z*‐scores and their associated standard errors for migraine and control participants for Adolescent/Adult Sensory Profile (AASP) and Hospital Anxiety and Depression Scale (HADS) subscales calculated from available normative means (where zero indicates the expected population mean, and 1 indicates one standard deviation above this for the population)

Of 944 participants, 117 (12%) scored 4 or above in the MS‐Q and were categorized as having probable migraine. Demographic details are presented in Table 1, and mean scores for both groups are presented as *z*‐scores in Figure 1, calculated using normative scores available for the AASP and HADS.^21^, ^34^

**TABLE 1:** Summary of demographic characteristics for both control and probable migraine participants

	Controls	Probable migraine
*N*	827	117
Mean age (SD)	53.6 (16.9)	48.3 (13.6)
No. female (%)	539 (65)	100 (85)

Between‐subjects *t*‐tests found that mean scores significantly differed between migraine and control participants for sensory sensitivity (*t*(942) = 8.05, *p* < 0.001, *d* = 0.80), sensory avoidance (*t*(942) = 7.24, *p* < 0.001, *d* = 0.71), and HADS‐A (*t*(942) = 8.78, *p* < 0.001, *d* = 0.87).

### Does anxiety mediate the sensory association with migraine?

**FIGURE 2:**
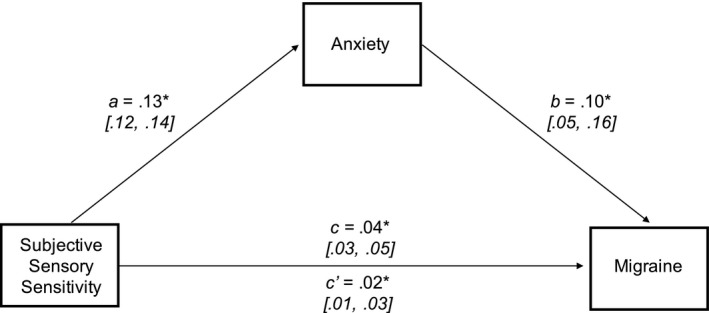
Mediation model of the relationship between subjective sensory sensitivity, anxiety, and migraine including 95% confidence intervals for each path. Each path denotes associations between variables of interest and is on a log‐odds metric. *p < 0.005

Mediation analysis was used to determine whether anxiety symptoms influenced the relationship between subjective sensory sensitivity and migraine (Figure 2). The total effect of subjective sensory sensitivity on migraine was significant (*c* = 0.04, *p* < 0.001). The estimated indirect effect via anxiety was 0.01, and the 95% bootstrapped confidence interval was entirely above zero (0.01–0.02), and thus significant. The direct effect of sensory sensitivity on migraine remained significant once this mediating effect was accounted for (*c*′ = 0.02, *p* = 0.001) indicating partial mediation.

Therefore, subjective sensory sensitivity was significantly associated with migraine both directly and via the mediating effect of anxiety symptoms. Note that mediation models are correlational and produce similar results if rotated (i.e., using subjective sensory sensitivity as the mediator). They do not establish causality.

### Sensory modality analyses

**FIGURE 3:**
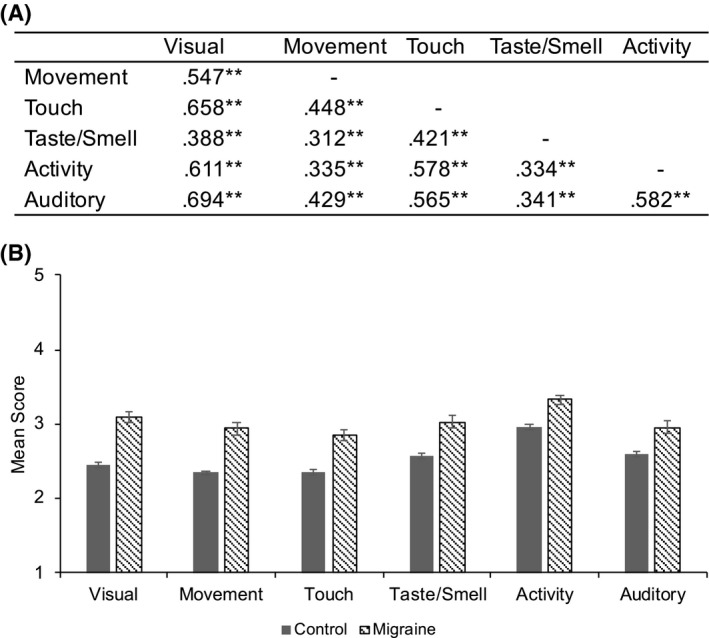
(A) Bivariate Pearson's correlations between each modality subscale taken from the Adolescent/Adult Sensory Profile (AASP), **p < 0.01. (B) Mean scores for each modality subscale for both migraine and control participants, standardized by dividing each mean by the number of items in the subscale

These exploratory analyses sought to determine whether the association between multisensory processing and migraine was driven by sensitivities in particular modalities. First, it is important to note that sensitivities in the different sensory modalities are correlated with each other (see Figure 3). However, all associated variance inflation factor values were below 5 or 10, the thresholds at which collinearity between variables is a concern^35^ (Visual = 2.81, Movement = 1.48, Touch = 2.08, Taste/Smell = 1.27, Activity = 1.86, Auditory = 2.14).

**FIGURE 4:**
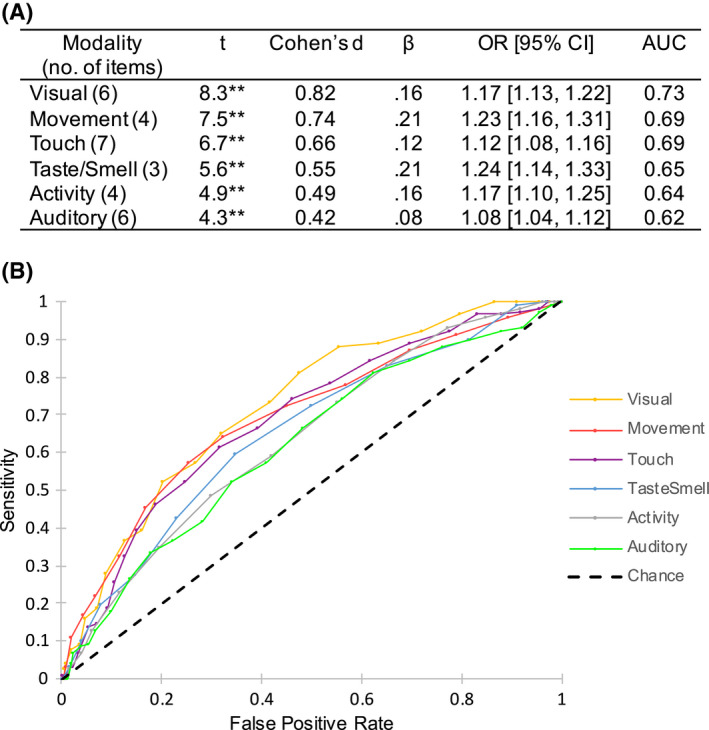
(A) A summary of *t*‐test and individual logistic regression analyses for modality subscales derived from the Adolescent/Adult Sensory Profile (AASP) predicting incidence of migraine, including area under the curve (AUC) values for each associated receiver operating characteristic curve displayed in Figure 4B. *p < 0.05, **p < 0.01. (B) Receiver operating characteristic curves for each modality subscale derived from the AASP, used to predict incidence of migraine

Given that each modality subscale was calculated using a different number of items (see Figure 4A), Figure 3 displays mean scores for each modality in a standardized form, calculated by dividing each raw mean by the number of items used to calculate that subscale.

Between‐subjects *t*‐tests were conducted to determine whether participants with migraine significantly differed in mean modality sensitivity scores when compared with controls. This was the case for all six modality subscales. Subsequently, individual logistic regression analyses found that each modality subscale significantly correlated with probable migraine (Figure 4).

Receiver operating characteristic curves and corresponding area under the curve values for each subscale are also displayed in Figure 4. Area under the curve values of greater than 0.55, 0.63, and 0.71 are thought to correspond to small, medium, and large effect sizes respectively (compared with Cohen's standards^36^). The majority of our modality subscales would thus be considered to have a corresponding medium effect size, with the exception of auditory and visual subscales, which have small and large effect sizes, respectively.

To determine the relative influence of each subscale, given that they correlate with each other, all six were included in a logistic regression model. The HADS‐A subscale was also included to control for its influence. As can be seen in Table 2, this model produced four significant predictor subscales: movement (*β* = 0.096, *p* = 0.010), visual (*β* = 0.105, *p* = 0.004), auditory (*β* = −0.071, *p* = 0.015), and HADS‐A (*β* = 0.104, *p* < 0.001). The Hosmer and Lemeshow test was nonsignificant for this model (*χ* ^2^ = 9.398, *p* = 0.310), suggesting that the model adequately fits the data.

**TABLE 2:** Summary of multivariate logistic regression analyses predicting incidence of migraine, using each modality subscale and anxiety as predictor variables

Modality	*β*	OR	95% CI	*p*
Constant	−5.05	0.01		<0.001
Visual	0.105	1.11	1.04, 1.19	0.004
Movement	0.096	1.10	1.02, 1.18	0.010
Touch	0.014	1.01	0.96, 1.07	0.614
Taste/smell	0.078	1.08	0.99, 1.18	0.090
Activity	−0.018	0.98	0.90, 1.08	0.697
Auditory	−0.071	0.93	0.88, 0.99	0.015
HADS‐A	0.104	1.11	1.05, 1.17	<0.001

All analyses were repeated removing participants who reported any vestibular conditions. The pattern of results remained the same and thus are not reported here.

## DISCUSSION

Sensitivities to sensory input are known to occur during migraine, but comparatively little is known about the extent to which such sensitivities continue between attacks. The present study therefore aimed to characterize whether individuals with migraine report higher interictal subjective sensory sensitivity across senses when compared with controls. In line with our hypothesis, this was found to be the case for both sensory sensitivity and sensory avoidance subscales of the AASP. We further hypothesized that this relationship would be at least partially mediated by symptoms of anxiety, given the co‐occurrence and commonalities in mechanisms seen in similar populations.^7^, ^14^, ^15^, ^16^, ^17^, ^37^ This second hypothesis was also supported. Possible causes and implications of these results are now discussed.

First, the finding that increased levels of subjective sensitivity to sensory input are associated with migraine aligns with and extends initial evidence of self‐reported sensory hypersensitivity in the condition.^10^, ^11^, ^12^, ^13^ Previous work in adults has used questionnaires designed to assess specific sensitivities (e.g., photophobia^13^), whereas this appears to be the first study to implement a broad, validated measure of subjective sensory sensitivity in this population. We found that these sensitivities are not limited to those inputs known to trigger migraine (e.g., light^13^), but instead significantly higher interictal sensitivity is observed across all sensory modalities.

Second, part of the relationship between subjective sensory sensitivities and migraine is accounted for by their relationships with anxiety symptoms. This is consistent with existing evidence that finds heightened levels of anxiety in those with sensory sensitivities.^9^, ^37^

However, as this study was cross‐sectional, further research is required to explore the direction of causality. Subjective sensory sensitivity may induce anxiety as the sensory input is perceived as overwhelming. Equally, input could provoke anxiety about an oncoming migraine attack in these groups, as sensory information is a cited migraine trigger.^2^ Alternatively, increased levels of anxiety may elicit a heightened reactivity to sensory stimuli.^38^ A causal mechanism such as this, stemming from anxiety, would be more readily amenable to treatment, particularly given that some pharmaceutical interventions show efficacy in treating both migraine and anxiety.^39^ Investigation of these relationships using a research design which allows for causal inference is needed. For example, determining whether reductions in anxiety relate to a reduction in subjective sensory sensitivity in this population could elucidate direction of effects. Importantly, information on participant's current medications was not collected in the current study; therefore, the possible influence of medication on these constructs could not be determined.

It is also important to note that although mediation effects were present, they did not entirely explain the relationship between subjective sensory sensitivity and migraine. A robust direct effect was still present, meaning even if anxiety symptoms were causative and were reduced through intervention, sensitivity might be expected to persist. The implications of interictal sensitivities therefore need to be understood and acknowledged in clinical management wherever necessary, with the awareness that these effects could vary across individuals. Biopsychosocial models of headache view pain and chronic illness as stemming from a complex interaction among biological, psychological, and social factors, with variation in these interrelationships contributing to differing illness presentations.^40^ In the context of migraine, anxiety may be more relevant to subjective sensory sensitivity in one person than another. Future work could thus build on these findings to determine how the presence of subjective sensory sensitivity and anxiety relates to migraine characteristics, such as frequency, severity, duration, and the presence of aura (which is known to relate to sensory sensitivities^41^, ^42^).

We also investigated whether sensitivities in certain sensory modalities were particularly important in predicting the incidence of migraine; visual, movement, and auditory subscales were significant predictors when controlling for scores in other modalities as well as anxiety. We had speculated that the visual domain may drive the main effects seen in our analyses, given that visual triggers and auras are commonly reported.^2^, ^4^

However, it is noteworthy that movement sensitivities were also significantly and positively predictive of migraine in these analyses. The movement subscale of the AASP assesses the presence of dizziness and avoidance or dislike of movement, which is known to be relatively common in those with migraine.^24^, ^43^ The association between movement sensitivity and migraine highlights how understanding sensory experiences in the condition could be beneficial in improving the current unmet need for nonpharmaceutical intervention. Physical activity is reported to reduce the severity of migraine, and yet those with migraine are found to exercise less regularly.^44^ Individuals with migraine may therefore need additional support to engage with exercise, with a focus on improving these sensitivities. Exercise may not only improve migraine, but also feelings of anxiety^45^ which independently, and via the influence on sensory sensitivities reported here, could further improve upon well‐being.

In contrast to the effects of the visual and movement subscales, the auditory subscale was significantly predictive of migraine but with a negative coefficient. This implies that a higher subjective auditory sensitivity is associated with reduced odds of migraine, which is counterintuitive in the context of auditory sensitivities and triggers.^2^ It is possible that this unexpected finding is merely statistical in nature, which can happen in a regression model where a notable amount of shared variance between factors exists, as is the case here. Additional work would be needed to determine the nature and role of subjective auditory sensitivity in migraine.

Lastly, for heightened sensitivity to touch and smell/taste, our results do not rule out their relevance for predicting migraine, but these contributions could not be disentangled from the correlations of these senses with vision, movement, hearing, and anxiety. Although widely used and validated, one limitation of the AASP is the limited number of items used to reflect taste and smell; of 30 questions assessing subjective sensory sensitivity, only three relate to taste/smell, and ultimately only one assesses olfactory sensitivity. Given this lack of clarity on the role of sensitivities in these modalities and the prominence of olfaction in migraine trigger literature,^2^, ^3^ future research could use initial findings reported here to more extensively explore subjective sensitivity in each modality independently using a measure with established modality subscales.

Enhancing understanding of modality‐specific sensitivities will also benefit from study which moves beyond focusing on only one form of sensory sensitivity, as the current literature tends to. For example, a combined approach that considers not only subjective sensory sensitivity but also sensitivity at a behavioral and neural level.^22^ It is not clear whether subjective sensory sensitivity and behavioral sensitivity are distinct; work relating the AASP to experimental sensory testing is largely focused on conditions such as autism spectrum disorder, and the results are mixed.^46^, ^47^ Recent work considering these relationships in the general population also finds that detection thresholds are not related to self‐reported sensitivity in either visual or auditory domains,^30^ and instead argues that they are distinct constructs. It is thus not known whether behavioral and subjective sensitivity would consistently co‐occur in the same individuals with migraine. Furthermore, combining questionnaire measures with neurophysiological data would allow us to relate subjective sensory sensitivities to existing models of cortical excitability^48^, ^49^, ^50^ to determine what underlies subjective sensory sensitivity at a neural level in this group.

Additional study limitations include the nature of recruitment; participants volunteered themselves after receiving an emailed advert, which potentially introduces self‐selection bias. Despite emphasizing the inclusivity of the survey in our recruitment advert in an attempt to mitigate this bias, this could explain why, for sensory sensitivity measures, our control participants scored slightly above normative data.

Participants were also not asked to confirm whether they were currently experiencing a migraine attack. However, the AASP does not ask about sensitivity in the current moment; instead, participants report the frequency with which each item is experienced. It is assumed that this would therefore represent the everyday, interictal experience. Additionally, it could be argued that individuals with migraine are unlikely to undertake a lengthy computer survey (as was required in the study) during an attack as this could exacerbate symptoms^51^ and there was no time limit to complete the survey.

Finally, as this was not an exhaustive exploration of possible correlates or mediators of sensory experiences in migraine, there are other factors that may also be relevant to relationships found here. For example, although anxiety has more established associations with sensory hypersensitivity, other traits, conditions, or clinical symptoms comorbid with migraine (e.g., neuroticism^52^) may also be relevant. How these parameters might affect the relationships described here would be an interesting avenue for future work for which this study can provide initial insight.

## CONCLUSIONS

In summary, interictal subjective sensory sensitivities were found to be significantly increased in migraine. This finding expands on extant literature by using validated questionnaire measures to consider sensitivities across several sensory modalities and additionally using a large community sample. We found that the relationship between subjective sensory sensitivity and migraine was partially mediated by anxiety symptoms. Although the causal mechanisms of this mediation are yet to be determined, this finding highlights the relevance of affect in sensory sensitivities between attacks. Targeting these symptoms therapeutically could improve upon sensory experiences, which may be affecting the quality of life and access to intervention in this population. Finally, it was found that visual and movement sensory sensitivities positively predicted the incidence of migraine, highlighting how these senses may be particularly important to the experience of sensory sensitivity in the disorder. Further investigation is needed to better understand the specific relevance of these modalities, perhaps with a focus on creating a unified understanding of sensory sensitivity across subjective, behavioral, and neural measures in migraine and beyond.

## CONFLICT OF INTEREST

All authors report no relevant conflict of interest.

## INSTITUTIONAL REVIEW BOARD APPROVAL

Institutional Review Board approval was granted by the Cardiff University's School of Psychology ethics committee.

## AUTHOR CONTRIBUTIONS

*Study concept and design*: Georgina Powell, Petroc Sumner. *Acquisition of data*: Georgina Powell. *Analysis and interpretation of data*: Alice Price, Petroc Sumner, Georgina Powell. *Drafting of the manuscript*: Alice Price. *Revising it for intellectual content*: Alice Price, Petroc Sumner, Georgina Powell. *Final approval of the completed manuscript*: Alice Price, Petroc Sumner, Georgina Powell.

## Supporting information

Fig S1

Fig S2

Fig S3

Supplementary Material
