# Supplementary figures and images for: Subjective sensory sensitivity and its relationship with anxiety in people with probable migraine
Source: Headache. 2021 Oct 20;61(9):1342–50. doi: 10.1111/head.14219 (PMC9889083; doi:10.1111/head.14219)

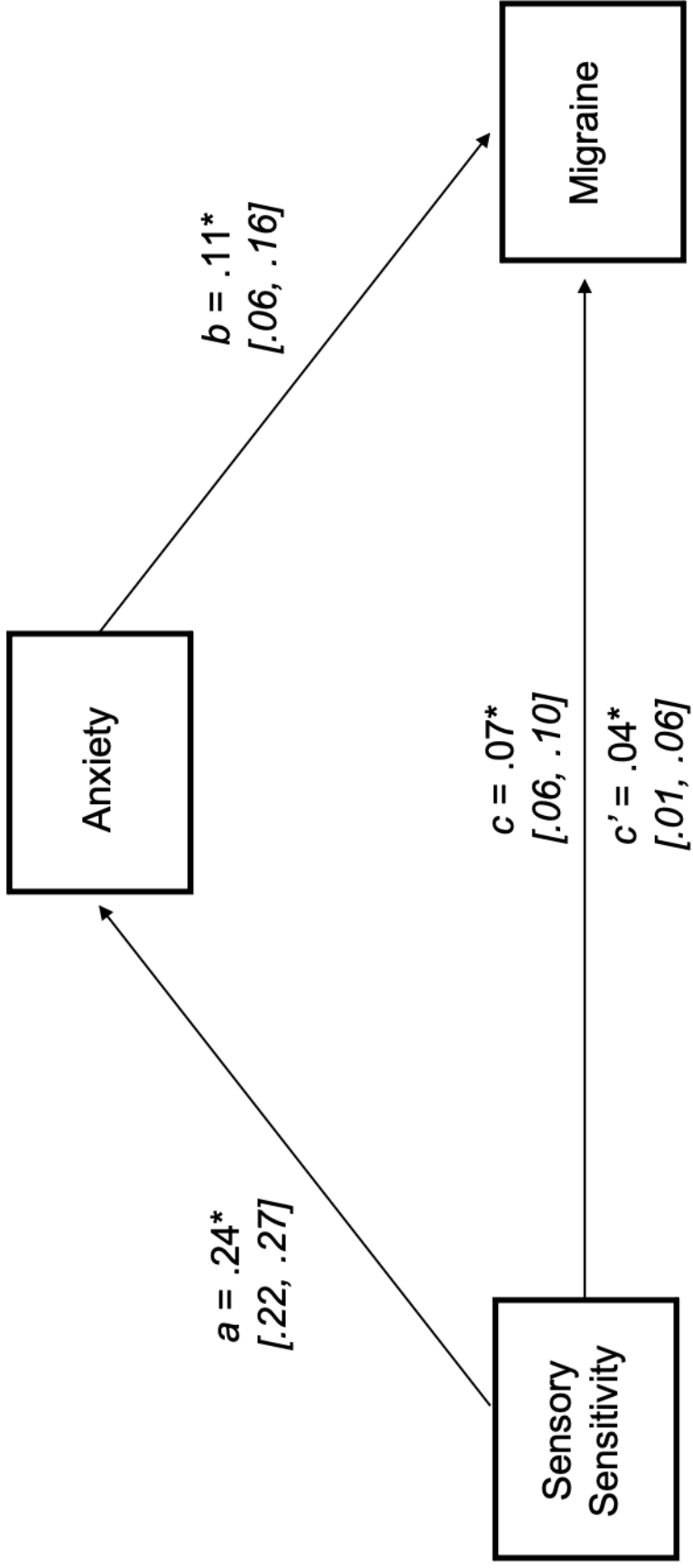

Supplement: Supplementary file 1 — Fig S1 [file HEAD-61-1342-s003.pdf]

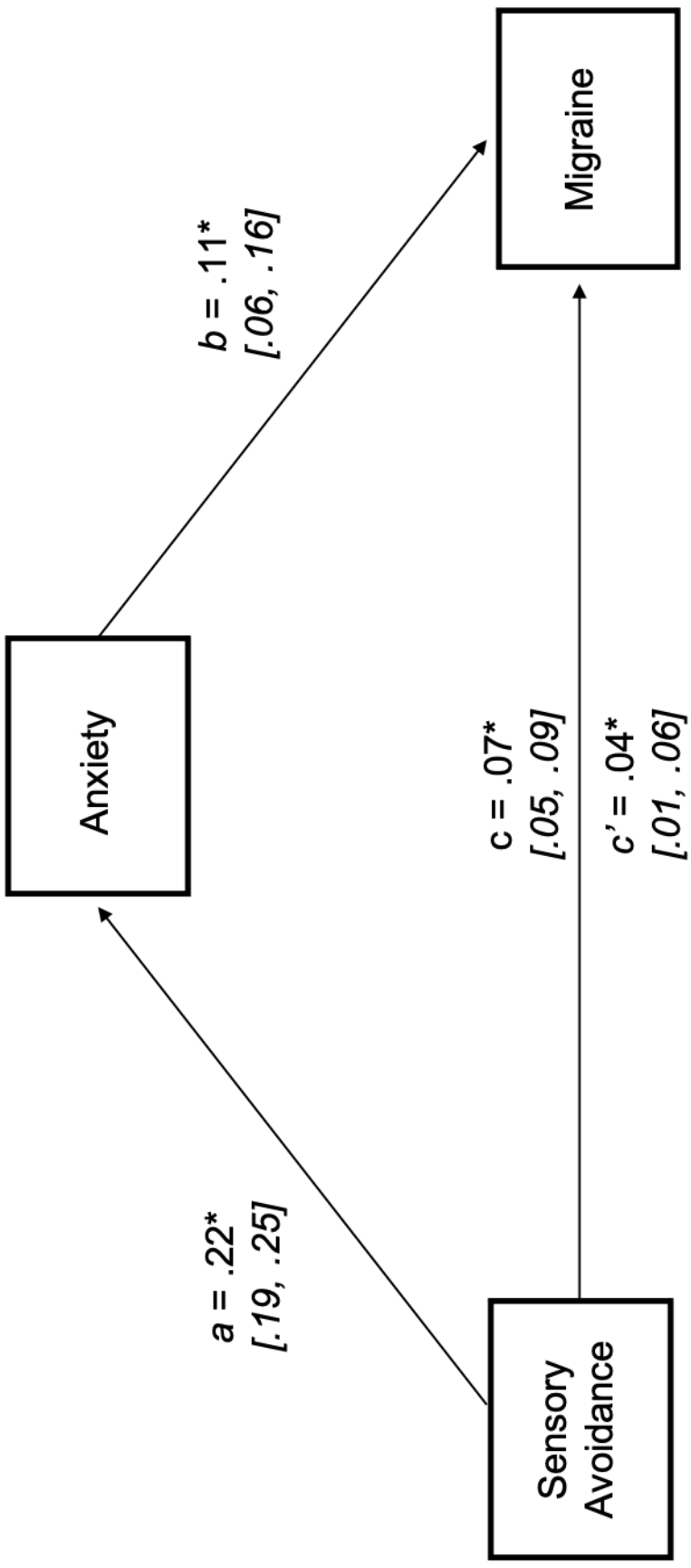

Supplement: Supplementary file 2 — Fig S2 [file HEAD-61-1342-s004.pdf]

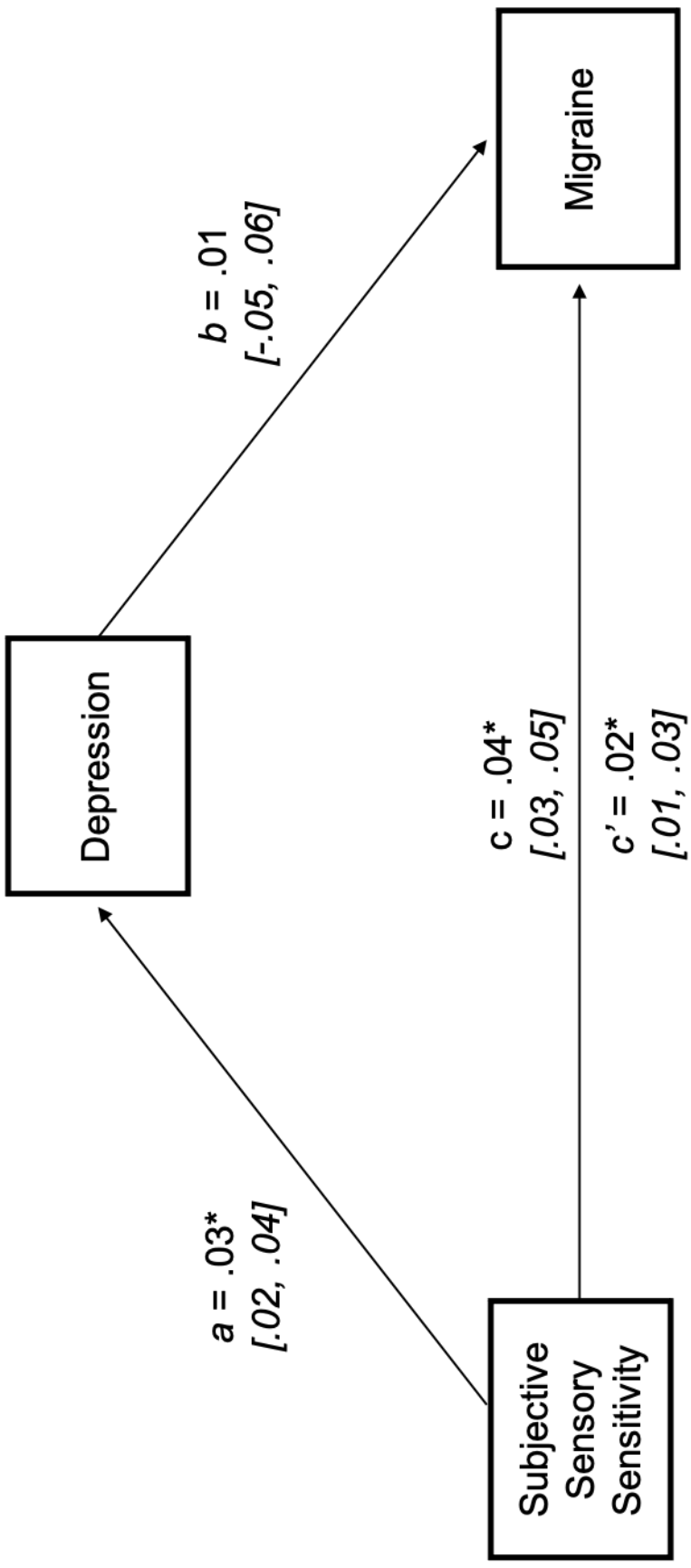

Supplement: Supplementary file 3 — Fig S3 [file HEAD-61-1342-s002.pdf]
