# Supplementary material for: Subjective sensory sensitivity and its relationship with anxiety in people with probable migraine
Source: Headache. 2021 Oct 20;61(9):1342–50. doi: 10.1111/head.14219 (PMC9889083; doi:10.1111/head.14219)
Supplement: Supplementary file 4 — Supplementary Material [file HEAD-61-1342-s001.docx]

Supplementary material

*Mediation analysis with separate AASP subscales*

Two mediation models were generated in the same manner as the main analyses, instead including sensory sensitivity and sensory avoidance subscales as the independent variable.

In the first mediation analysis, we considered whether anxiety symptoms influenced the relationship between the sensory sensitivity subscale and migraine (Figure S1). The total effect of sensory sensitivity upon migraine was significant (c = .06, p < .001). The estimated indirect via anxiety was 0.03, and the 95% bootstrapped confidence interval was entirely above zero (0.01 to 0.04), and thus significant. The direct effect of sensory sensitivity upon migraine remained significant once this mediating effect was accounted for (c’ = .04, p = .002), indicating partial mediation.

*[Figure S1]*

Similarly, the total effect of sensory avoidance upon migraine was significant (c = .06, p < .001). The indirect effect of anxiety was 0.02, which is also significant (0.01 to 0.04). The direct effect of sensory avoidance upon migraine remained significant (c’ = .04, p = .003). These effects are displayed in Figure S2 and suggest the presence of partial mediation.

*[Figure S2]*

To summarise, both sensory measures (sensory sensitivity and sensory avoidance) were significantly associated with migraine both directly, and via the mediating effect of anxiety symptoms.

*Mediation analysis using depression symptoms*

Given that anxiety was found to be a significant partial mediator in our initial analyses, it was of interest to determine whether depression symptoms similarly influenced the relationship between subjective sensory sensitivity and migraine.

Depression symptom scores in our participants with probable migraine are indeed significantly higher than our control sample (t (942) = 7.20, p < .001, d = 0.71), a known association in the literature^52^. However, in a mediation analysis (covarying for age, gender and anxiety symptoms), depression was found not to mediate the relationship between sensory sensitivity and migraine (Indirect effect = .002, LLCI = -.003, UCLI = .006), supporting our hypothesis that depression does not influence this relationship in our sample. These findings are summarised in Figure S3.

*[Figure S3]*

*Figures*

**Fig S1** *Mediation model of the relationship between sensory sensitivity, anxiety and migraine including 95% confidence intervals for each path. Each path denotes associations between variables of interest and are on a log-odds metric. *p < .005.*

**Fig S2** *Mediation model of the relationship between sensory avoidance, anxiety and migraine including 95% confidence intervals for each path. Each path denotes associations between variables of interest and are on a log-odds metric. *p < .005.*

**Fig S3** *Mediation model of the relationship between subjective sensory sensitivity, depression and migraine including 95% confidence intervals for each path. Each path denotes associations between variables of interest and are on a log-odds metric. *p < .001.*
